# Effects of facilitatory and inhibitory Kinesio taping on lateral gastrocnemius muscle activity, motor neuron excitability, and countermovement jump height in university athletes from multiple sports: A randomized controlled trial

**DOI:** 10.1016/j.heliyon.2023.e23230

**Published:** 2023-12-04

**Authors:** Ahmar Raza, Saima Zaki, Md Farhan Alam, Saurabh Sharma, Tuba Aysha, Ahmad Talal Khiyami, Ayman Jaber Althobaiti, Hani Awwadh Alnefaie, Shibli Nuhmani

**Affiliations:** a Centre for Physiotherapy and Rehabilitation Sciences, Jamia Millia Islamia, Maulana Mohammad Ali Jauhar Marg, New Delhi, 110025, India; b Department of Physical Therapy, King Abdulaziz Hospital, Makkah Saudi Arabia; c Department of Physical Therapy, College of Applied Medical Sciences, Imam Abdulrahman Bin Faisal University, Dammam, Kingdom of Saudi Arabia

**Keywords:** Kinesiology taping, Muscle activity, Motor neuron excitability, Vertical jump, Electromyograph

## Abstract

**Objectives:**

This study aims to investigate the temporal effects of two Kinesio Taping (KT) techniques on lateral gastrocnemius muscle activity, motor neuron excitability, and countermovement jump height in university athletes from hockey, football, basketball, and volleyball. Additionally, it investigates whether the athletes' playing positions—either attacker or defender—influence these outcomes following the KT application.

**Methods:**

Forty-eight subjects were randomly assigned to one of three groups: Group A (n = 16), Group B (n = 16), and Group C (n = 16). All groups were further subdivided into attackers and defenders. Adhesive Kinesio tape was applied to the lateral gastrocnemius using the Y-shaped technique for three days. Facilitatory KT was applied from the origin to the insertion of the lateral gastrocnemius, while inhibitory KT was applied from the insertion to the origin. Motor neuron excitability, electromyographic activity, and countermovement jump height were tested at baseline, as well as after KT application, to evaluate if the dependent variables had changed. One-way ANOVA was used for baseline comparison, and mixed ANOVA was applied to assess post-interventional effects on the outcome measures.

**Results:**

Significant group effects for lateral gastrocnemius activation were found, measured using percentage of maximum voluntary isometric contraction (% MVIC) average root mean square (RMS). In motor neuron excitability, maximal M-wave (M_max_) was significantly improved in group comparison. Further, there was also a significant increase in countermovement jump height. There was no significant difference in outcome measures based on playing position (attacker and defender).

**Conclusion:**

Both KT techniques effectively influenced the lateral gastrocnemius muscle's activity, motor neuron excitability, and jump height when compared with the control group. Additionally, there is no effect of playing position, specifically attacker or defender, on the examined variables following KT application.

## 1 Introduction

Kinesio Taping (KT), since gaining prominence in the 2008 Olympic Games, has attracted considerable attention from sports practitioners and physiotherapists as a potential tool for injury prevention and performance enhancement [1]. It is designed to assist the body's intrinsic healing mechanisms by providing both support and stability to muscles and joints without the restricting joint range of motion [2]. Particularly relevant in sports rehabilitation, KT serves a dual purpose: it not only helps alleviate pain and facilitate lymphatic drainage by microscopically lifting the skin but also plays a critical role in re-educating the neuromuscular system [1]. The application of KT is meticulously tailored to individual muscular patterns, aiming to either re-educate or stabilize specific body regions, thereby enhancing strength and range of motion [1,3].

Elucidating its foundational principles, the design of this elastic tape is crafted to optimize skin tissue and neuromuscular function [4]. The direction of its application—from a muscle's origin to its insertion or vice versa—determines its facilitative or inhibitory effects [5]. Proponents of KT claim that it can offer both facilitatory and inhibitory effects on the underlying musculature, depending on the technique of application [6]. These effects have the potential to alter muscle activity and neuromuscular responses, which can consequently impact sports-related movements such as the countermovement jump (CMJ) [7,8]. The CMJ is a robust measure of an athlete's lower body explosive power, incorporating a rapid downward movement succeeded by a maximal vertical jump [9,10], any intervention that can influence CMJ height could offer a competitive edge to athletes. This concept is further supported by empirical studies like those of Sinaei et al. (2021) and MacDowall et al. (2015), which have analyzed the impact of KT on muscle activity and athletic performance, particularly in tasks like vertical jumping [11,12]. Such insights demonstrate the pivotal role of KT in sports science, which provides both therapeutic and performance-enhancing benefits.

Yet, the efficacy of KT in influencing physiological parameters and enhancing performance remains debatable. For instance, while Sinaei et al. (2021) found that electromyography (EMG) amplitude of vastus medialis oblique activity increased and pain intensity decreased post-KT, other studies such as Briem et al. (2011) and Serrão et al. (2016) reported no significant alteration in muscle activity due to KT application [[12], [13], [14]]. While Słupik et al. (2007) demonstrated a positive effect of KT on quadriceps peak torque and EMG activity [15]. A previous study have compared the effects of KT to no tape on isometric strength and EMG activity of plantar flexor muscles in 24 healthy participants [16]. When the foot was fully dorsiflexed, they noticed an increase in strength and EMG activity [16]. Another study has provided supporting evidence for KT by demonstrating significant increase in vertical ground reaction force and EMG activity of the medial gastrocnemius during the jumping task when compared with control [17]. In contrast another previous study found no significant difference in jump height following the KT intervention [11] While studies on motor neuron excitability, which is determine by amplitude of Hoffman reflex through stimulating type-Ia afferent sensory fibers [18], have provided further evidence on effect of KT. For example, studies by Amin Kordi Yoosefinejad et al. (2016) and Firth et al. (2010), which both evaluated motor neuron excitability post-KT but found no immediate changes [19,20]. Such discrepancies could be attributed to factors like variations in study design, KT application techniques, or participant demographics. Therefore, the available literature on KT as an adjunct to enhance muscular performance or injury rehabilitation is limited and presents conflicting findings, underscoring the need for more comprehensive and standardized research to determine its efficacy reliably.

While there is existing literature on the effects of KT on muscle activity, motor neuron excitability, and vertical jump performance, no study has collectively analyzed these parameters by comparing effects of both facilitatory and inhibitory KT with a control group. Therefore, the present study aims to comprehensively assess the effects of both facilitatory and inhibitory KT on muscle activity, motor neuron excitability, and CMJ height performance in university athletes. Additionally, this study aims to evaluate whether the playing positions—specifically as an attacker or defender—influence EMG activity, motor neuron excitability, and CMJ height following KT application in these athletes. Based on these objectives, we hypothesized that both facilitatory and inhibitory KT will have distinct effects on muscle activity, motor neuron excitability, and CMJ height performance in university athletes, with potential differences observed based on playing positions. The attackers and defenders might display varying neuromuscular attributes influenced by the application of KT due to their unique roles in gameplay [21,22]. The findings from this study hold the potential to guide tailored injury prevention and performance enhancement strategies based on KT techniques and playing positions. Furthermore, it may refine current taping protocols in sports physiotherapy, merging academic insights with practical training methodologies.

## 2 Materials and methods

### 2.1 Research design

The study was designed as a parallel-group, multiple-arm, randomized controlled trial and is registered in the Clinical Trials Registry-India (CTRI) with the registration number [CTRI/2023/02/049,387]. The researcher adheres to the values stated in the Helsinki Declaration of 1964 [23]. This study was submitted for ethical approval from institutional human ethical committee, Jamia Millia Islamia and was approved (28/11/419/JMI/IEC/2022). The participants have provided their signed informed consent before enrolment in the study, with their consent for photography for this study. The dependent variables were motorneuron excitability, EMG activity and countermovement jump height. The independent variable was KT.

### 2.2 Sample size calculation

We utilized G*Power software version 3.1.9.4 (Franz F, Universität Kiel, Kiel, Germany) to determine the necessary sample size for our study. Through comprehensive theoretical discussions, our research team concluded that an a priori large effect size assumption of f = 0.5 is justified given the expected outcomes, informed by an extensive review of the relevant literature and our collective expertise. Setting the alpha level at 0.05 and the power threshold at 85 %, we determined a sample size of 48 participants (16 per group) would be required to ensure statistical significance (*p* < 0.05). In accordance with the Intention-to-Treat (ITT) principles, our sample size estimation has not anticipated drop-out rate. Our approach commits to the inclusion and analysis of all participants who are randomized at the outset of the trial. Any instances of missing data were addressed using appropriate imputation techniques. This methodological rigor maintains the integrated of our randomized trial design.

### 2.3 Participants

A sample of 48 university athletes was recruited from the Sports Complex at Jamia Millia Islamia University, New Delhi. The sample comprised hockey, football, basketball, and volleyball players actively involved in sport-specific training at least two days per week.

The inclusion criteria were as follows: aged between 18 and 26 years, both males and females, participating in one of the aforementioned sports, and regularly engaged in sport-specific training for a minimum of two days per week. Participants were excluded if they had a history of lower limb fractures; any pathology affecting the spine, hip, knee, or foot within the past six months; leg length discrepancy; allergies to adhesive tape; rheumatological or neurological disorders; neurogenic low back pain within the past six months; or alcohol addiction, which could potentially affect the H-reflex or myofascial trigger points in the lateral gastrocnemius muscle.

**Fig. 1:**
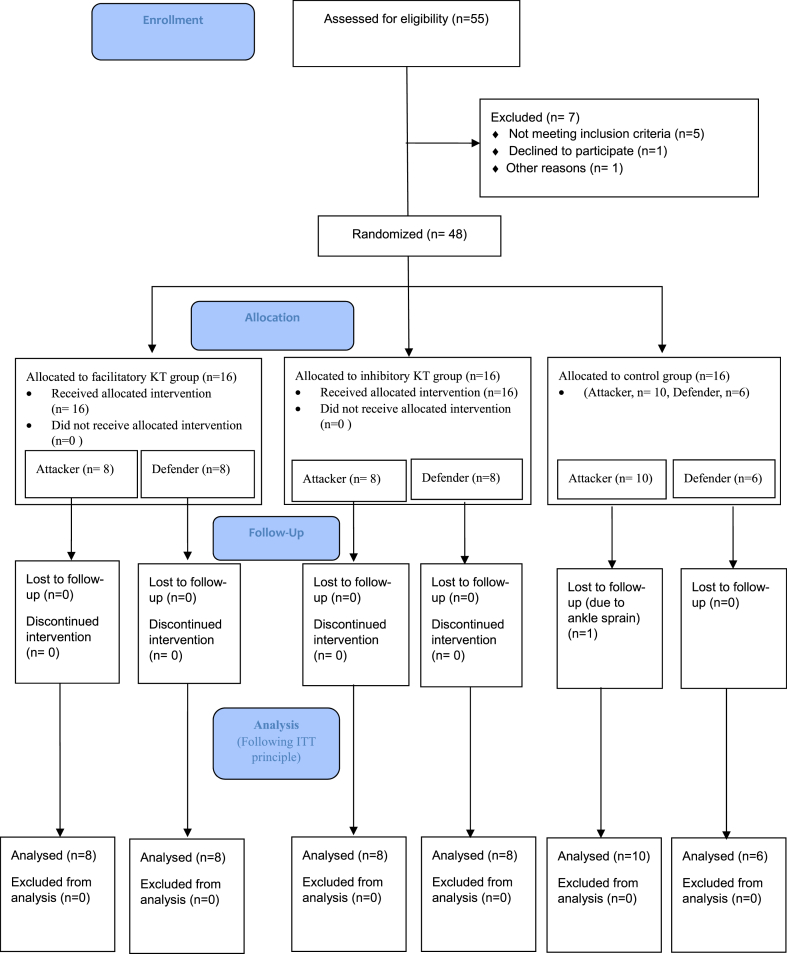
Flow diagram showing participants in each phase of the study.

Participants were allocated to one of three groups using block randomization with a block size of six, ensuring an even distribution across the groups during the enrollment phase. Each block included two participants for Group A (facilitatory KT technique), Group B (inhibitory KT technique), and Group C (daily routine training, no KT). The sequence within each block was determined using a computer-generated random sequence, employing the RAND function in Microsoft Excel. This method guaranteed an equal number of participants in each group by the end of the enrollment period. The randomization was solely for group assignment and was not applied when categorizing participants into attacker or defender roles within the groups (Fig. 1).

After group allocation, baseline assessments of motor neuron excitability, EMG activity, and countermovement jump height were conducted. These measures were then reassessed after 3 days of KT application to determine any changes in the dependent variables. Participants and outcome assessors were blinded to the type of KT received, to minimize potential placebo or expectation effects, ensuring an unbiased evaluation of the results. Although the assessor applying the tape was aware of the group assignments, this knowledge was essential due to the distinct techniques required for each taping method. To maintain the integrity of the study, this assessor was not involved in the subsequent evaluation of outcomes.

### 2.4 Evaluation

#### 2.4.1 Anthropometric measurement

Height (m) of the subject was measured using wall mounted stadiometer with subject standing against the wall and feet closed together without shoes. Weight (kg) of the subject was measured using weighing machine. Leg dominance was determined using Ball Kick Test (BKT), the subject was asked to kick a soccer ball with moderate intensity and maximal accuracy through a set of cones placed 1 m apart and 10 m from the subject. Three trials of this test were conducted. The leg use to kick the ball was identified as the dominant leg [24].

#### 2.4.2 Countermovement jump

The countermovement jump (CMJ) began with the athletes standing and lowering fast to a self-selected position then quickly extending their hip, knee, and ankle joints to attain a maximal jump height [25]. Participants were given instructions to jump vertically and perform a maximal vertical jump while actively swinging their arms. The maximum height of the jump was measured at the fingertip with a measuring tape attached to white paper on which each subject's finger ink print was clearly recorded. This procedure was repeated three times, and the average jump height was documented.

#### 2.4.3 Electromyography

**Fig. 2:**
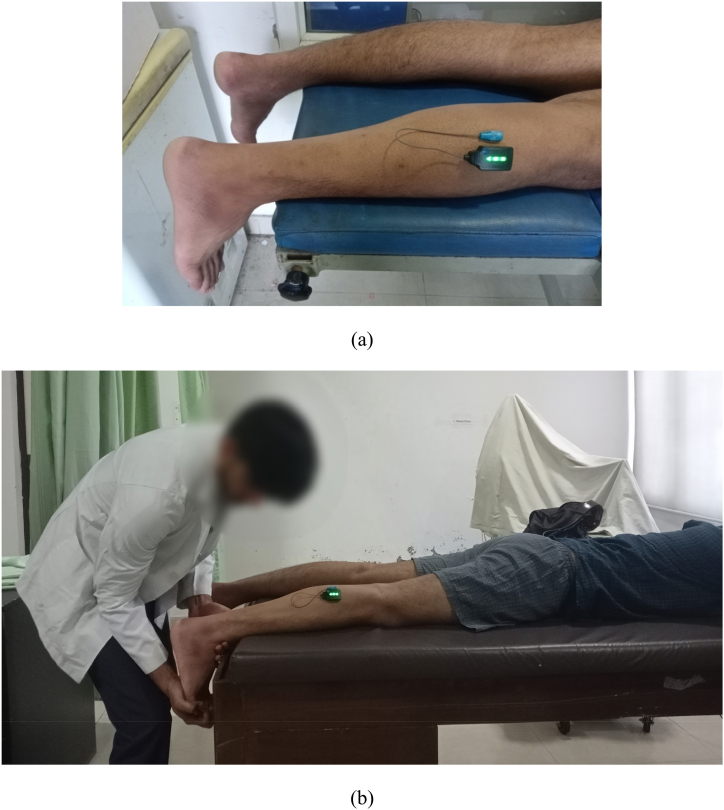
Maximum voluntary isometric contraction measurement of lateral gastrocnemius: (a) surface electrode placement for electromyographic recording, (b) subject is performing maximum voluntary isometric contraction.

EMG activity of the lateral gastrocnemius was recorded using the Delsys Trigno wireless EMG system and Lab Chart software version 7, AD instruments, New Zeland. A surface EMG sensor was used to connect individuals to the wireless surface EMG System. The skin of each sensor location was prepared and cleansed prior to sensor placement to reduce electrical impedance. The subject lay prone with the dominant knee extended and foot extending over the end of the table for land marking of the sensor site of lateral gastrocnemius. Before sensor placement, a reference mark was drawn on each participant's skin to ensure the sensor could be accurately repositioned for the assessment three days later. Subjects were advised not to wash off the mark to ensure it remained visible for the follow-up measurement. Additionally, the same trained investigator, familiar with the study's assessment protocol and the participant's anatomy, was responsible for both the initial placement and the subsequent repositioning of the sensor. This approach was taken to ensure the uniformity of our procedures and to minimize potential variability in EMG readings due to sensor placement. The sensor was placed on the lateral head of the gastrocnemius on the participant's dominant leg, one-third of the distance between the fibula head and the calcaneus, the sensor pointing in the direction of the muscle fibers as per the Surface Electromyography for the Non-Invasive Assessment of Muscles (SENIAM) guidelines [Fig. 2 (a)] [26]. After applying the EMG electrodes, the subject performed a practice countermovement jump to ensure that the sensor was securely connected, functional, and not affecting his or her performance.

The MVIC (Maximum Voluntary Isometric Contraction) measurements for the lateral gastrocnemius were taken to normalize the EMG amplitude and calculate the percentage of MVIC (% MVIC). To determine the MVIC, subjects were positioned prone with their knees extended and feet overhanging the end of the table. Each participant performed three 5-s MVICs through plantar flexion of the ankle against manual resistance [Fig. 2 (b)]. After the MVIC testing, subjects were given a 5-min rest period. Subsequently, subjects were instructed to perform the CMJ to calculate the Root Mean Square (RMS). They completed three trials of the movement, taking a 10-s rest between each trial.

Percentage MVIC calculation-

% MVIC = (RMS/MVIC) × 100

#### 2.4.4 EMG signal processing

Muscle activity data, recorded with wireless EMG sensors, were sampled at 2000 Hz from lateral gastrocnemius muscle of the dominant leg using a wireless electromyography (EMG) system (Delsys Trigno wireless EMG system, New Zealand). The raw EMG data were band-pass filtered ranging from 10 to 500 Hz, full wave rectified, and then the root mean-square (RMS) of the signal was derived.

#### 2.4.5 H-reflex

**Fig. 3:**
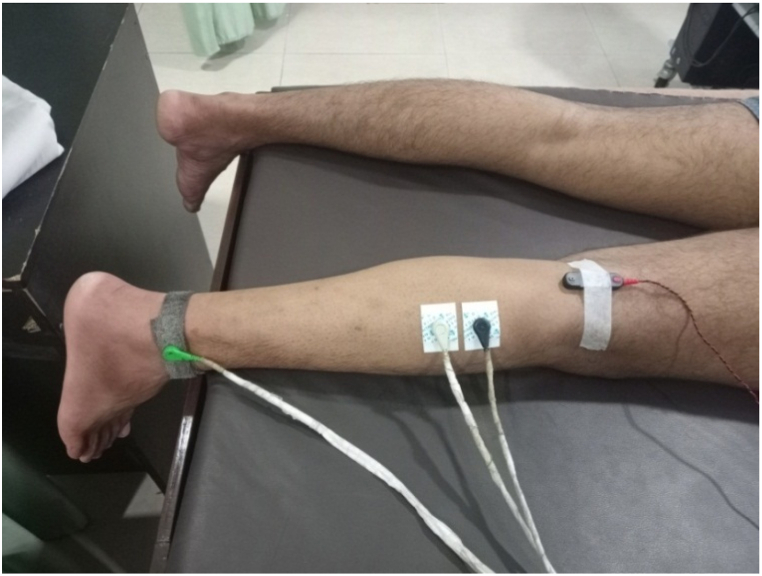
Surface electrode placements for H-reflex recording.

The shaved/hairless skin was cleansed with alcohol swab to help reduce skin impedance. The H-reflex was recorded with an EMG system (Power Lab 15T, AD instrument, Labchar-7, Australia). The tibial nerve was stimulated with stimulating bar electrode placed in the middle of the popliteal fossa [27]. H- and M-waves were recorded from the lateral head of the gastrocnemius muscle using surface electrodes placed according to the Surface Electromyography for Non-Invasive Assessment of Muscles (SENIAM) guidelines—one-third of the distance between the head of the fibula and the calcaneus [26]. Hmax, or the maximal H-reflex, is the highest amplitude of the H-reflex that can be elicited from the muscle, indicating the peak excitability of the spinal motor neurons. M_max_, or maximal M-wave, is the largest compound muscle action potential resulting from direct motor nerve stimulation and represents the maximal response of the muscle fibers [28]. A dry earth strap was used for grounding and was placed over the lateral malleolus. To ensure proper placement of the stimulating electrode, it was initially moved at a slower pace to locate the tibial nerve in the popliteal fossa. Upon determining this location by evoking a response in the calf muscles, the stimulating electrode was secured with micropore tape. The duration of each stimulus was 1 ms, and the intensity was gradually increased to obtain H_max_ and M_max_, as well as the ratio of H_max_ to _Mmax_ (H/M ratio) (Fig. 3.).

### 2.5 Intervention

**Fig. 4:**
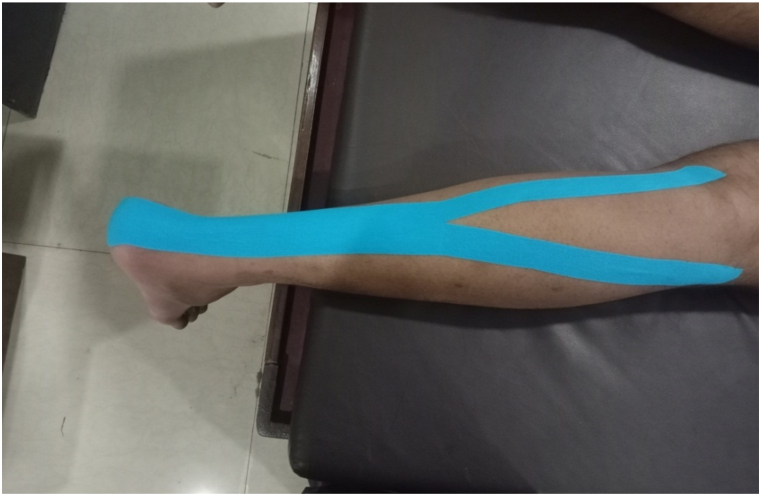
Y-shaped kinesiology tape application.

Adhesive waterproof KT 5 cm wide and 0.5 mm thick was used in this study. The participants’ dominant leg was shaved from the knee down to increase the adhesion of the tape and then the subjects were positioned in prone, with the dominant foot completely dorsiflexed and the calf muscles stretched. The tape was cut by estimating the distance between the distal end of the plantar aspect of the calcaneus and the distal end of the gastrocnemius muscle belly and then each of the four corners rounded. The tape was applied by an experienced physiotherapist to the lateral gastrocnemius using a Y-shaped technique as proposed by Ref. [29]. The proximal and distal ends of the tape were applied under no tension while the foot was in a neutral position. The Y-shaped technique was used to either facilitate or inhibit muscle [29]. Facilitatory KT was applied to the leg from the origin to the insertion of the lateral gastrocnemius at 50 % tension, while inhibitory KT was applied from insertion to origin at 15 % tension [30].The tape was then activated by rubbing it for 10 s. The tape was applied for 72 h in both groups [29] (Fig. 4.).

**Fig. 5:**
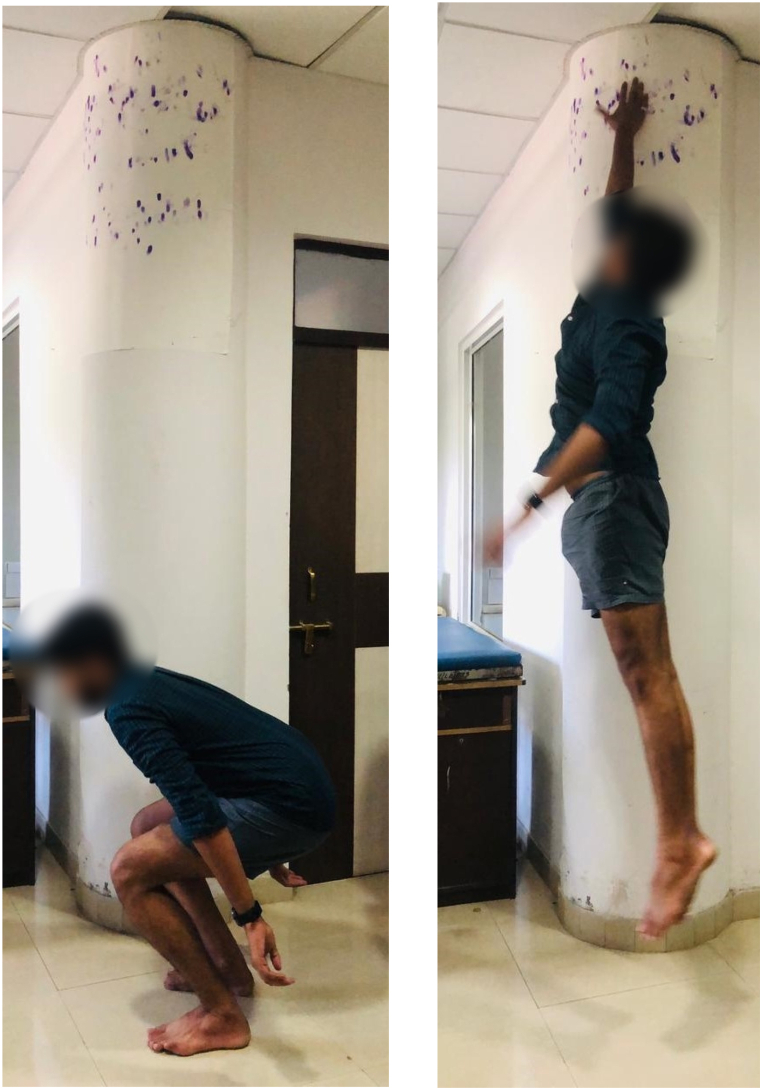
Subject performing countermovement jump.

## 3 Statistical analysis

All data were analyzed using SPSS version 27 (SPSS Inc., Chicago, Illinois). The mean and standard deviation (SD) values are used to represent data. The data were evaluated for the normality of the distribution scores using the Shapiro-Wilk test. The demographic characteristics and the baseline criterion measures were compared between the three groups – facilitatory KT (group A), inhibitory KT (group B) and control (group C) - by one-way analysis of variance (ANOVA). Further comprehensive evaluation of the post-intervention data was achieved using repeated measures ANOVA. The ANOVA enabled us to discern the main effects of time, indicating overall changes in the measurements over the intervention period; the main effects of group, identifying if there were systematic differences in outcomes between the taping strategies and control; and the main effects of playing position, determining if attackers and defenders experienced different results. We also investigated interaction effects such as Time × Group, Time × Playing Position, and Group × Playing Position to identify if the changes over time or the differences between groups varied according to the specific conditions of the other factors. Bonferroni's post hoc were employed to identify the differences between groups when a significant difference was observed. The paired-t test was applied to analyse difference between baseline and post-intervention. Significance level was set at *p* < 0.05.

## 4 Results

Initially, 48 athletes from football, hockey, volleyball, and basketball were recruited for our randomized controlled trial. Although one participant from the control group was unable to continue due to an ankle sprain, we retained the full sample size in our analysis. This was achieved by applying the ITT principle and utilizing the Last Observation Carried Forward (LOCF) method, thereby including data from all 48 participants in the final evaluation to maintain the integrity of the randomized design**.**

### 4.1 Demographic characteristics and baseline data

The data was checked for normality, and all variables were found to be normally distributed.

All three groups were comparable at baseline since there were no statistically significant differences (*p* > 0.05) in any of the outcome variables. (Table 2.). All the groups were comparable in terms of age, height, weight, BMI (Table 1).

**Table 1:** Demographic characteristics of participants for Group A (facilitatory kinesiotaping group), Group B (inhibitory kinesiotaping group) and Group C (no taping group).

	Group-A	Group-B	Group-C
*n*	16	16	16
Age	22.43 ± 2.6	21.62 ± 1.5	23.12 ± 1.8
Gender (M:F)	11 M: 5F	13 M: 3F	11 M: 5F
BMI (kg/m^2^)	22.49 ± 2.07	21.03 ± 1.7	22.30 ± 2.2
Leg dominance (R:L)	14 : 2	13 : 3	12 : 4
No.of strikers (n)	8	8	10
No.of defenders (n)	8	8	6
Height (m)	1.72 ± .07	1.72 ± .0.85	1.67±.o8
Weight (kg)	66.43 ± 7.95	65.43 ± 8.3	62.25 ± 12.5

Abbreviations: *n*: number of participants; M: male; F: female; BMI: body mass index; kg: kilogram; m: meter, R: right, L: left.

**Table 2:** Baseline comparison of participants for Group A (facilitatory kinesiotaping group), Group B (inhibitory kinesiotaping group) and Group C (no taping group).

Variables	Attacker (n = 26)			Defender (n = 22)			Difference due to group (*p*-Value)	Difference due to playing position (*p*-Value)
	Group A (n = 8)	Group B (n = 8)	Group C (n = 10)	Group A (n = 8)	Group B (n = 8)	Group C (n = 6)		
Avg MVIC (mv)	0.14 ± .09	0.11 ± .07	.13 ± .06	0.12 ± .06	0.11 ± .05	0.09 ± .06	0.62	0.30
Avg RMS (mv)	.53 ± .25	.52 ± .20	.41 ± .20	.56 ± .23	.69 ± .25	.55 ± .35	0.28	.093
% MVIC (normalized RMS)	424.35 ± 135.47	425.04 ± 134.8	347.94 ± 122.73	449.04 ± 141.96	474.06 ± 122.97	368.16 ± 196.55	0.12	0.32
Hmax (mv)	.12 ± .08	.18 ± .12	.19 ± .1	.22 ± .07	.20 ± .07	.20 ± .09	0.78	0.15
Mmax (mv)	.18 ± .07	.20 ± .11	.21 ± .07	.16 ± .06	.24 ± .19	.12 ± .02	0.34	0.63
H/M ratio	.36 ± .21	.30 ± .13	.23 ± .13	.30 ± .11	.33 ± .21	.31 ± .12	0.46	0.51
CMJ height (cm)	259.38 ± 14.31	254.02 ± 13.90	261.02 ± 15.70	266.02 ± 22.90	262.76 ± 9.06	244.02 ± 18.90	0.41	0.88

Abbreviations: n: number of participants, mv: millivolts, RMS: root mean square, cm: centimeter, MVIC - maximum voluntary isometric contraction; CMJ-countermovement jump; H/M ratio - ratio of maximum H_wave_ amplitude and maximum M_wave_ amplitude.

**Table 3:** Post-intervention comparison of participants for Group A (facilitatory kinesiotaping group), Group B (inhibitory kinesiotaping group) and Group C (no taping group).

Variables	Attacker (n = 26)			Defender (n = 22)			Time (T) ɳp^2^ (p-Value)	Group (G) ɳp^2^ (p-Value)	Playing position (PP) ɳp^2^ (p-Value)	T × G ɳp^2^ (p-Value)	T × PP ɳp^2^ (p-Value)	G × P ɳp^2^ (p-Value)	T × G × PP ɳp^2^ (p-Value)
	Group A (n = 8)	Group B (n = 8)	Group C (n = 10)	Group A (n = 8)	Group B (n = 8)	Group C (n = 6)							
Avg MVIC (mv)	0.37 ± 0.33	.25 ± .27	.14 ± .07	.25 ± .23	.19 ± .25	.09 ± .06	.169 (<.01**)	.116 (.07)	.04 (.17)	.09 (.12)	.018 (.39)	.003 (.94)	.008 (.83)
Avg RMS (mv)	.81 ± .18	.48 ± 0.27	.32 ± .17	.90 ± .45	.80 ± .54	.23 ± .11	.015 (.43)	.30 (<.01**)	.06 (.09)	.242 (<.01**)	.000 (.96)	.05 (.32)	.04 (.38)
% MVIC (normalized RMS)	558.50 ± 185.47	466.82 ± 175.42	295.8 ± 140.01	555.68 ± 201.99	369.80 ± 168.86	368.80 ± 196.57	.01 (.48)	.21 (<.01**)	.002 (.76)	.12 (.06)	.01 (.49)	.01 (.73)	.04 (.39)
Hmax (mv)	.15 ± .03	.20 ± .13	.16 ± .10	.24 ± .10	.20 ± .08	.18 ± .07	.003 (.73)	.005 (.90)	.05 (.14)	.073 (.20)	.001 (.82)	.05 (.33)	.008 (.84)
Mmax (mv)	.49 ± .10	.52 ± .34	.19 ± .09	.65 ± .32	.72 ± .39	.13 ± .03	.51 (<.01**)	.39 (<.01**)	.01 (.37)	.35 (<.01**)	.05 (.12)	.08 (.15)	.01 (.71)
H/M ratio	.40 ± .23	.36 ± .15	.24 ± .13	.35 ± .12	.32 ± .17	.32 ± .13	.08 (.05)	.04 (.39)	.001 (.87)	.03 (.48)	.01 (.47)	.03 (.49)	.03 (.48)
CMJ height (cm)	262.65 ± 13.87	264.81 ± 7.89	247.01 ± 17.98	273.38 ± 12.89	268.23 ± 8.44	250.38 ± 18.11	.02 (.26)	.24 (<.01**)	.01 (.42)	.07 (.17)	.02 (.27)	.08 (.16)	.07 (.19)

Abbreviations: n: number of participants, mv: millivolts, RMS: root mean square, cm: centimeter, MVIC - maximum voluntary isometric contraction; CMJ-countermovement jump; H/M ratio - ratio of maximum H_wave_ amplitude and maximum M_wave_ amplitude, T: time, G: group; PP: playing position.

### 4.2 *Post-intervention* outcomes (Fig. 5 and Table 3.)

#### 4.2.1 Average MVIC

**Fig. 6:**
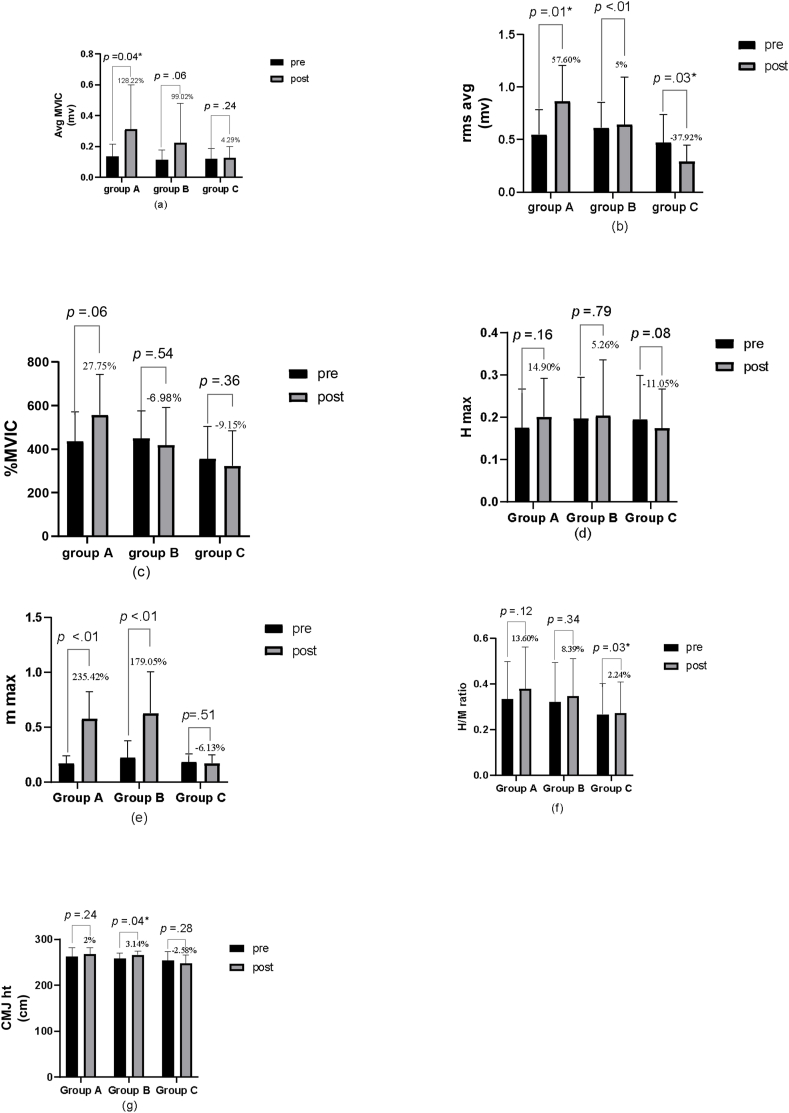
Mean, standard deviation and percentage change of pre-test and post-test outcome measures: a) average maximum voluntary isometric contraction (MVIC); (b) average root mean square (RMS); (c) percentage MVIC; (d) Hmax; (e) Mmax (f) H/M ratio; (g) countermovement jump height.

Following 3 days intervention of KT the ANOVA revealed significant effect of time (*p* < 0.01). While the main effect of group was insignificant as well as main effect of playing position, interaction of Time × Group, Time × playing position, Group × Playing position), Time × Ggroup × Playing position was also insignificant. Within-group analysis revealed that Group A (128.22 %) and Group B (99.02 %) had improved more than Control Group C (4.29 %) [Fig. 6 (a)].

#### 4.2.2 Average RMS

ANOVA yielded significant effect of group (*p* < 0.01) and interaction of Time × Group (*p* < 0.01). While all other variables were found to be insignificant. The results of the within-group study revealed that Group A (57.60 %) had improved more than Group B (5 %). While we noted a decline in group C of 37.92 % [Fig. 6 (b)].

#### 4.2.3 % MVIC

ANOVA yielded significant effect of Group (*p* < 0.01). Rest of all shown insignificant. It was revealed that Group A EMG activity increased by 27.75 % while Group B activity declined by 6.98 % and Group C by 9.15 % [Fig. 6 (c)].

#### 4.2.4 Maximum amplitude of H-wave (Hmax)

ANOVA revealed insignificant effect of Time, Group, Playing position and as well as interaction among them was not significant. The percentage mean difference was found to be 14.90 % in group A, 5.26 % in group B and a reduction of 11.05 % in group C [Fig. 6 (d)].

#### 4.2.5 Maximum amplitude of M-wave (Mmax)

Significant Group (*p* < 0.01), time (*p* < 0.01), and interaction Group × Time) effects (*p* < 0.01) were found. While all the parameters related to playing position were found to be not significant. Within group analysis demonstrated a significant improvement in both the intervention groups (Group A- 235.42 %, Group B- 179.05 %). While in group C we have noted a reduction of 6.13 % [Fig. 6 (e)].

#### 4.2.6 H/M ratio

ANOVA yielded insignificant effect of time, group, playing position and interaction of ime × Group, ime × playing position, Group × Playing position and Time × Group × Paying position. Within group analysis revealed a 13.60 % improvement in group A, 8.39 % in group B and 2.24 % in group C [Fig. 6 (f)].

#### 4.2.7 Counter movement jump height

Significant group (*p* < 0.01) effect was found; however, effect of time, playing position and interaction of Time × Group, Time × Playing position, Group × Playing position), (Time × Group × Playing position were found to be insignificant. While within group analysis revealed 2 % improvement in group A, 3.14 % in group B and a reduction of 2.58 % in group C [Fig. 6 (g)].

## 5 Discussion

In the evolving landscape of sports science and rehabilitation, KT has emerged as a promising tool to modulate neuromuscular activity and optimize athletic performance. The present study was designed to investigate the effect of different KT techniques on motor neuron excitability, muscle activation, and functional performance in university athletes. The findings of the present study revealed that type of taping significantly influenced certain neuromuscular and performance parameters, regardless of the athlete's specific playing position. Such universal applicability underscores the potential of KT as a versatile intervention for diverse athletic profiles.

### 5.1 Effect of KT on muscle activation

In our randomized controlled trial of 48 athletes, there was a significance differences between groups following the application of KT in muscle activity, assessed by % MVIC and average RMS. However, the average MVIC showed a significant main effect of time. This suggests that irrespective of the taping technique applied, there were changes in MVIC over time, possibly due to factors such as muscle fatigue, adaptability, or the placebo effect of the taping itself [31,32]. Furthermore, the facilitatory KT group (Group-A) demonstrated a substantial mean difference (MD) in % MVIC which was 27.75 %. These findings are in line with Kase and Wall's (2013) concept that indicated distinct outcomes according to the direction of KT application [28]. It is apparent from these findings that the nature of KT application—be it facilitatory or inhibitory—dictates its impact on muscle function and activity. Given that these metrics provide insights into muscle activation and strength, it can be inferred that the facilitatory and inhibitory taping techniques differently modulate muscle activation patterns. This aligns with previous literature suggesting that KT can alter afferent feedback, muscle recruitment strategies, and thereby, the overall muscle activation [29].

Consistent with our results, a previous study have demonstrated that KT application significantly elevated the EMG activity of the medial gastrocnemius during jump tasks, further substantiating our findings [17]. More recent evidence from Zaworski, K. et al. (2022), highlighting a significant increase in gluteus medius activity following a 48-h facilitatory KT application [33]. Furthermore, studies assessing the effects of inhibitory KT techniques offer complementary insights. For example, Martínez-Gramage, J. et al. (2016), reported a shortened duration of lateral gastrocnemius activity following inhibitory KT application, aligning with the reductions in muscle activity we observed in Group-B [34].

The underlying mechanisms proposed for these observations are multifaceted. Yam M L. et al. (2019) suggest that the force generated by the recoiling action of the KT may be transmitted to the fascia, thereby aiding muscle contractions, especially when the directions of muscle contraction and KT pull are congruent [6]. This could be the reason for higher muscle activation observed in the facilitatory KT group. Conversely, for inhibitory KT techniques, if the direction of the muscle contraction opposes the pull of the KT, it is theorized that the KT's pull may activate the Golgi tendon organs, potentially inhibiting muscle contraction [6]. This might explain the reduced EMG activity post-inhibitory KT application.

### 5.2 Effect of KT on motorneuron excitability

The effect of KT on motor neuron excitability was examined in this study and compared to a control group without tape. The amplitude of the Hoffman reflex (H-reflex) is a measuring unit to determine motor neuron excitability, by stimulating type-Ia afferent sensory fibers [18]. Such stimulation results from direct synapsing of these fibers onto alpha motor neurons in the spinal cords anterior horn [18,35]. Stimulating this arc produces a compound muscle action potential termed as H wave, which depends on the balance of excitatory and inhibitory neurons in the spinal cord [28]. Predominantly recorded from the gastrocnemius and soleus muscles, this reflex can be stimulated electrically, and its response captured using electromyography (EMG) as the H wave [18,36]. Normalizing the H-reflex can be achieved by dividing the maximum H-reflex (H_max_) amplitude by the maximum M-wave (Mmax) amplitude (H/M ratio), where a lower ratio indicates motor neuron inhibition and a higher ratio suggests excitation [37]. In our study we have examined H_max_, M_max_ and their ratio as a outcome measures of motor neuron activity.

The current study has shown that following KT intervention the H_max_ remained unchanged. This is consistent in line with the previous study which have reported that calf muscles motor neuron excitability remained unaltered following the application of the kinesiology tape [19]. However, the KT method used in their study was a tendon correction technique [19]. [30]. Expanding on this, Yoosefinejad et al. (2017) undertook a comprehensive exploration into the neuromodulatory effects of KT. Their findings were parallel to ours, suggesting that neither facilitatory nor inhibitory taping methods had a substantial impact on the amplitude of the H-reflex [30]. Consistency in unchanged H_max_ post-KT in previous studies and in our findings suggests that the Ia afferent input into the alpha motor neuron remained unaltered, indicating that KT might not have a strong modulatory effect on these primary afferent inputs. Additional studies exploring diverse KT application methods, a broader range of muscle groups, and more specific assessments of neuromuscular function could provide greater understanding on the exact mechanisms underlying KT's influence.

Furthermore, the M_max_, revealed a significant main effect for both time and group differences. This finding underscores the potential of KT in modulating the neuromuscular system at the motor neuron level [38,39]. In line with our findings, prior studies have also shown that the application of KT can enhance electrical activity and the recruitment of motor neurons in the quadriceps muscle [15]. Further, statistical analyses demonstrated that there was no significant change in motorneuron excitability in terms of H/M ratio between the groups. Although, MD of within the group was higher in facilitatory KT (13.60 %) as compared with inhibitory KT group (8.39 %).

The potential mechanisms underlying the impact of KT on motor neuron excitability can be multifaceted and may involve a combination of physiological and neurological influences. One of the primary proposed mechanisms is that KT might alter afferent feedback to the central nervous system by mechanically lifting the skin, which in turn affects the sensory neurons that are involved in the monosynaptic reflex arc of the H-reflex [29,38]. The lifting of the skin could change the interstitial pressure and improve the flow of blood and lymphatic fluids, thereby potentially influencing the metabolic environment of the motor neurons [29]. Moreover, KT might have a mechanical impact that leads to a change in muscle tension. This alteration could modulate the length-tension relationship of the muscle spindles, thus affecting the Ia afferent input to the alpha motor neurons and subsequently the excitability of the motor neuron pool [40]. The facilitatory or inhibitory effects of KT could then be hypothesized to result from these changes in afferent input, although the precise neurophysiological pathways remain to be fully elucidated.

### 5.3 Probable mechanisms underlying the pronounced effect size in average RMS measurements and myogenic response

In the study examining the effects of facilitatory and inhibitory KT relative to a control group, the variables % MVIC and average RMS, indicative of muscle activation, displayed high effect sizes. The substantial effect sizes in % MVIC and average RMS suggest that KT might have a considerable impact on muscle fiber recruitment and the level of muscle activation [6]. This could result from KT's mechanical action on the skin, potentially enhancing proprioceptive feedback and promoting increased motor unit activation rates, which would be reflected in heightened EMG activity, as indicated by these metrics [6,41].

The M_max_ variable also exhibited a significant effect size both across time and between groups, which implies that KT may influence the motor neurons' ability to generate robust action potentials. This could be due to changes at the neuromuscular junction or modifications in the overall excitability of the motor neuron pool [42]. The greater impact on M_max_, as opposed to H_max_, might signify that KT's modulatory effects extend to influencing the intrinsic properties of motor neurons and their action potential generation capabilities, rather than solely affecting synaptic transmission or reflex pathways as indexed by the H-reflex [18]. These findings underscore the notion that KT's neuromodulatory actions are multifaceted, with pronounced effects at both the muscle activation and motor neuron excitability levels.

### 5.4 Effect of KT on countermovement jump height

In our study, the analysis of CMJ height revealed statistically significant group differences. Participants receiving facilitatory KT (Group A) exhibited a 2 % MD in jump height, whereas those with inhibitory KT (Group B) showed a 3.14 % MD improvement. Such findings suggest that different KT applications may have specific influences on the neuromuscular system and mechanical properties of muscles and tendons, which could account for the observed changes in CMJ height [7,43].

The impact of KT on CMJ performance has been debated, with various studies yielding contradictory results. Investigations by Huang et al. (2011) and MacDowall et al. (2015) found no significant enhancements in CMJ height immediately after KT application This absence of immediate effects was also observed by Boozari et al. (2018). These studies suggest that the benefits of KT, if any, might not be instantaneous or may vary depending on the protocol used [8,11,17]. In contrast, our study aligns with the findings of Mendez-Rebolledo et al. (2018), where an improvement in jump height was noted, particularly after 72 h of KT application [44]. This suggests a potential delayed effect of KT on CMJ performance, implicating a temporal factor in the efficacy of KT. The three-day duration, consistent with our study's protocol, provides a temporal window within which KT might exert its effects, possibly due to prolonged neuromuscular facilitation or other underlying mechanisms that evolve over time [44].

A multifaceted interaction between mechanical and neuromuscular factors is revealed in the mechanism of KT to enhance CMJ performance. For the facilitatory KT, the enhancement in vertical jump height may be partially due to an improved pattern of muscle activation. By potentially augmenting the muscle spindle's response, the application of facilitatory KT might amplify the neuromuscular system's ability to respond to rapid muscle fiber stretching and the propagation of vibrational waves through tissue. This, in turn, could trigger a more pronounced active stretch reflex, affecting both short- and long-latency reflex pathways [45,46]. This neuromuscular adjustment could then lead to the recruitment of additional motor units or an increase in the firing rate of active units, leading to enhanced force production during both the eccentric and concentric phases of the CMJ [47].

In contrast, the improvement in CMJ height following inhibitory KT may operate through a different mechanism. The inhibitory taping technique may decrease muscular tension and potentially increase muscle length, possibly allowing for a greater range of motion [29,48]. Such changes could enhance the musculotendinous structures' ability to store and release elastic energy more effectively during the CMJ [49]. The pre-stretching effect caused by this taping could facilitate the storage of elastic energy, which is then released during the explosive concentric action of the jump, thus contributing to improved jump performance [14,29,50].

Together, these effects suggest that KT can enhance CMJ performance by influencing both neuromuscular activation and the biomechanical properties of the musculoskeletal system. Notably, the specific mechanisms through which KT exerts its effects may vary according to the technique of application. This complexity inherent in KT's impact highlights its versatility and potential for customization to improve athletic performance. By tailoring the application of KT, practitioners can potentially optimize its efficacy, thereby aiding athletes in achieving superior outcomes in their respective fields. Further research could elucidate the precise biomechanical and neurophysiological changes induced by these taping methods to fully understand their role in enhancing jump performance.

## 6 Effect of KT on playing position

Our study aimed to investigate the impact KT on distinct playing positions, specifically focusing on attackers and defenders. This differentiation was based on the hypothesis that the distinct roles and activities performed by these positions might manifest differences in their neuromuscular response to KT application. However, our results showed no significant variation in any of the measured variables, including muscle activity, motor neuron excitability, or countermovement jump height due to playing position post-KT application. This indicates that the influence of KT, regardless of being facilitatory or inhibitory, is consistent across these positional distinctions.

The underlying causes for these findings may be attributed to the physiological workings of KT. It's postulated that KT augments muscle performance by means of sensory stimulation, enhancing blood circulation, and promoting lymphatic drainage [1,2]. These effects, being largely superficial, might not be deeply influenced by the specific muscle usage or movement patterns associated with either playing position. Consequently, both attackers and defenders, despite having different game-play responsibilities, might experience similar sensory feedback and circulatory effects from the tape, leading to comparable outcomes in the measured parameters. While these insights provided understanding regarding our findings, more detailed studies are necessary to further explore how KT functions in relation to the specific requirements of attackers and defenders during gameplay.

To the best of our knowledge, our study is novel in its approach, being the first to compare the effects of KT in different playing positions. Previous research has largely focused on the application of KT across various sports or its effects on specific injuries without necessarily dissecting its effects based on the intricacies of playing positions within a sport [1,51]. Drawing from these insights, the implications of our findings gain added importance for sports practitioners and therapists. If KT effects do not significantly differ between playing positions, this provides a uniform framework for its application across a team. Therapists can simplify team settings by not modifying their taping techniques or expecting different outcomes based on an athlete's on-field role.

## 7 Limitations

This study presents several limitations worth noting. Primarily, the lack of data immediately following the KT application constitutes a notable drawback. Gathering this data could have shed light on the initial impacts of KT, offering a more holistic understanding of its temporal dynamics. Additionally, while the study investigated the effects of KT on the lateral gastrocnemius muscle, its exclusive focus on this specific muscle poses challenges to generalizability. The insights, while valuable, might not be directly applicable to other muscle groups, necessitating caution in extrapolation. Another limitation could be the non-inclusion of alternative muscle strength assessment measures, such as the handgrip dynamometer, manual muscle testing, and ground reaction force analysis. Incorporating these measures could have enriched our comprehension of KT's effects. Furthermore, our study did not incorporate a follow-up phase, limiting our understanding of the sustainability and long-term effects of KT. Given these considerations, professionals and researchers are encouraged to judiciously apply our findings, especially when considering other anatomical or functional contexts.

## 8 Strength

Despite the above limitation, the present study has following potential strength. Firstly, the study adhered to standardized protocols, which aimed to maintain methodological consistency throughout the research process. Secondly, by included both genders in our sample, our research addresses the diverse physiological responses and effects of KT, enhancing the robustness of our findings. Thirdly, the usage of high-quality, premium-grade tape in the study helps ensure the findings are not skewed by varying material quality, thus supporting the reliability of our observations. Lastly, the differential effects of KT across various playing positions are a novel addition to the existing literature, providing a unique perspective on KT's potential role in sportspersons with different roles and responsibilities.

## 9 Future prospective

Future investigations would be enriched by incorporating multiple KT sessions, extending the follow-up periods, and monitoring the longer-term effects and adaptations. Such prolonged observations would offer a more thorough understanding of the sustained impacts and possible advantages of KT interventions. Moreover, undertaking a dose-response study of KT would be prudent. The potential psychological effects of KT may be successfully separated and measured by integrating placebo taping alongside KT application. These methodological improvements would provide deeper insights into KT's real physiological impact, enabling a better knowledge of its therapeutic efficacy and processes.

## 10 Conclusion

In conclusion, the findings of this study suggest that the application of both facilitatory and inhibitory KT techniques significantly enhances the activity of the lateral gastrocnemius muscle, motoneuron excitability, and CMJ height when compared to a no-tape control group. Additionally, the study found no significant effect of playing position, whether attacker or defender, on the variables measured post-KT application. These findings highlight the potential benefits of KT as a modality for improving muscular and neuromotor function in athletes irrespective of their playing positions.

## Data availability statement

The data that support the findings of this study are available on request from the corresponding author, Dr. Saurabh Sharma. The data are not publicly available due to privacy and ethical restrictions. The subjects participating in this research did not consent to have their data publicly shared, and the study design was approved by the Institutional Ethical Committee, Jamia Millia Islamia (28/11/419/JMI/IEC/2022), with the understanding that the data would remain confidential.

## CRediT authorship contribution statement

**Ahmar Raza:** Writing – original draft, Methodology, Investigation, Data curation, Conceptualization. **Saima Zaki:** Methodology, Investigation, Formal analysis, Data curation, Conceptualization. **Md Farhan Alam:** Writing – original draft, Project administration, Methodology, Investigation, Formal analysis. **Saurabh Sharma:** Writing – review & editing, Validation, Supervision, Project administration, Formal analysis, Conceptualization. **Tuba Aysha:** Resources, Methodology, Investigation, Formal analysis, Data curation. **Ahmad Talal Khiyami:** Writing – review & editing, Writing – original draft, Resources, Formal analysis. **Ayman Jaber Althobaiti:** Writing – review & editing, Validation, Resources, Formal analysis. **Hani Awwadh Alnefaie:** Writing – review & editing, Writing – original draft, Validation, Supervision, Resources. **Shibli Nuhmani:** Writing – review & editing, Supervision, Resources, Project administration, Formal analysis.

## Declaration of competing interest

The authors declare that they have no known competing financial interests or personal relationships that could have appeared to influence the work reported in this paper.
